# Be up and Doing

**Published:** 1897-03

**Authors:** 


### BE UP AND DOING!

Legislative efforts on every hand are awakening the people to new conditions and a fresh appreciation of the needs of the times; and there is something for each of us to do. There must be many minds and many hands to do the work that is needed; there must be a quickening of our zeal and a prompt entering upon the work of the hour. There is legislation at Washington to aid the army veterinarians. There is legislation in Maine to better guard the interests of veterinarians; other proposed methods to better direct State veterinary sanitary work. There is legislation in New Hampshire and in Connecticut to deal with both these subjects. There are needed legislative enactments in Massachusetts to eliminate features of the law regulating the Cattle Commission, in default of which there will be no further real progress in the Bay State. There are laws to be secured in New York; there are other proposed ones to defeat. In Pennsylvania the enlarging of the powers and means of the State Veterinary Sanitary Board and a better protection of our borders from the introduction of untested animals should be secured. Virginia seeks more power in State sanitary police measures. Louisiana looks forward to the same. North Carolina is eager to get in line for recognition of her professional representatives. New Jersey, Michigan, Minnesota, Wisconsin, Illinois, and Colorado are all seeking stronger safeguards for their animal industries and for recognition of those who have given time and money to prepare themselves for the profession. All this means much hard work; something for all to do in aiding every one of these efforts. Your voice in State Association meetings—your pen in the press or to your friends and acquaintances or through kindred organizations—is needed to aid this strong movement spreading over the land. So we appeal to you to be up and doing, and when the evening of 1897 shall have been reached there will be common cause for all to rejoice in the returning prosperity and bright future of our profession. SPEED THE GOOD WORK !

